# Diagnostic accuracy of tablet-based software for the detection of concussion

**DOI:** 10.1371/journal.pone.0179352

**Published:** 2017-07-07

**Authors:** Suosuo Yang, Benjamin Flores, Rotem Magal, Kyrsti Harris, Jonathan Gross, Amy Ewbank, Sasha Davenport, Pablo Ormachea, Waleed Nasser, Weidong Le, W. Frank Peacock, Yael Katz, David M. Eagleman

**Affiliations:** 1 Dalian Medical University, Dalian, Liaoning Province, China; 2 Department of Emergency Medicine, Baylor College of Medicine, Houston, Texas, United States of America; 3 BrainCheck, Inc, Houston, Texas, United States of America; 4 Department of Neuroscience, Baylor College of Medicine, Houston, Texas, United States of America; 5 Department of Psychiatry & Behavioral Sciences, Stanford University School of Medicine, Stanford, CA, United States of America; University of Florida, UNITED STATES

## Abstract

Despite the high prevalence of traumatic brain injuries (TBI), there are few rapid and straightforward tests to improve its assessment. To this end, we developed a tablet-based software battery ("BrainCheck") for concussion detection that is well suited to sports, emergency department, and clinical settings. This article is a study of the diagnostic accuracy of BrainCheck. We administered BrainCheck to 30 TBI patients and 30 pain-matched controls at a hospital Emergency Department (ED), and 538 healthy individuals at 10 control test sites. We compared the results of the tablet-based assessment against physician diagnoses derived from brain scans, clinical examination, and the SCAT3 test, a traditional measure of TBI. We found consistent distributions of normative data and high test-retest reliability. Based on these assessments, we defined a composite score that distinguishes TBI from non-TBI individuals with high sensitivity (83%) and specificity (87%). We conclude that our testing application provides a rapid, portable testing method for TBI.

## Introduction

### Background

Between 1.6 and 3.8 million cases of traumatic brain injuries (TBI) are reported every year in America [1], of which 75% are classified as mild (mTBI) [2]. A large fraction of these cases come from military warzones, as well as from team sports such as football, rugby, hockey and soccer [3]. Collectively, these injuries result in over 50,000 deaths, leave over 70,000 patients with permanent neurological problems, constitute the leading cause of death and disability of U.S. children and young adults, and cost approximately $60B annually [2]. Further, it is a growing belief that the number of TBI cases are underestimated due to the lack of a central reporting system and the fact that many people with less severe injuries do not seek medical treatment [1, 4, 5]. Such statistics and the downstream neurodevelopmental consequences [6] have created a call to action to provide more objective measures of cognitive functioning—both before an injury occurs, as well as post-injury to measure recovery [7].

### Importance

Despite the prevalence of mTBI, early diagnosis of the condition remains a challenge [8]. Many individuals with mTBI do not immediately exhibit symptoms and do not have neurological deficits that can be detected by brain imaging or mental state examination. It is estimated that only about 10% of TBIs are detected by CT scans where brain bleeding has occurred, leaving 90% to other diagnostic criteria. Thus, while moderate and severe TBI are fairly easily diagnosed, mild TBI often escapes notice. Unfortunately, early detection of mTBI is critical to avoid secondary damage, which is sometimes irreversible [9].

### Goals of this investigation

Although early detection of mTBI is often missed [1], it does not have to be. Subtle brain injury can be detected with tests of attention, perception, and visuomotor skills [10]. We set out to determine whether such tests could be adapted to a tablet device and be used to detect mTBI. To that end, we developed a tablet-based software battery with the aim of maximizing diagnostic accuracy, portability, and ease of operator use, while minimizing testing time and the possibility of malingering. Our approach capitalizes on brief; simple tests appropriate for tablet computing to tease out several aspects of mTBI.

Our primary research aim is to estimate the diagnostic accuracy of BrainCheck when compared to physician diagnoses of concussion. We also sought to determine the distribution of performance for each test variable, identify differences in performance driven by age or sex, quantify test-retest reliability, and evaluate the efficacy of a software module intended to test for malingering.

## Methods

### Selection of participants

This study was approved by the Institutional Review Board of the Baylor College of Medicine, and each participant was required to provide written informed consent. Informed verbal accent was required for adolescent participants in addition to written informed consent from their guardian.

#### TBI population and pain-matched controls

Eligible subjects were patients admitted to the Emergency Department (ED) at Ben Taub Hospital with a chief complaint of head trauma, and were determined by a physician evaluation to have suffered a mTBI in accordance with the diagnostic criteria [11]. We also enrolled pain-matched control subjects from the ED, defined as those clinically determined to be experiencing similar amounts of pain but without any head injury (e.g. a twisted ankle). Physicians were blind to our test results because they reached diagnoses immediately prior to the administration of BrainCheck.

#### Normal population

For comparison, we also enrolled a healthy control population. Data was gathered from universities, high schools, community centers, and the Texas Medical Center accelerator (specifically, Rice University, University of Houston, Houston Community College, St. Thomas High School, Emery Weiner High School, KIPP Generations, KIPP Houston, KIPP Sunnyside, Tellepsen YMCA, Trotter YMCA, Weekly YMCA, Robinson Judson Jr. Community Center, Fonde Community Center, and the Texas Medical Center accelerator. Retest data was only collected from the Texas Medical Center accelerator). Participants from the local universities and the TMC accelerator were administered the test one-on-one; participants from the high schools and community centers were administered both individually and also in groups of 2–12 people at a time. To ensure quality participant effort, each testing site had a minimum of three test administrators.

#### Inclusion and exclusion criteria

All participants were required to be between the ages of 18 and 64, have full function of both hands and perfect or corrected vision. For the mTBI group, inclusion criteria were as follows: patients were required to have presented to the ED with a suspected traumatically-induced brain injury (if the patient was not sure that the head was directly injured in the traumatic event, they needed to report a loss of consciousness [LOC] or amnesia); patients were required to have a Glasgow Coma score (GCS) of 14 or 15 on initial evaluation in the ED; patients or their legal representatives had to be willing to undergo the Informed Consent process prior to enrollment into the study. Additionally, we required a physician diagnosis of mTBI in accordance with the diagnostic criteria of mTBI [11].

Subjects were excluded from the study if any of the following criteria were true: TBI within the last 6 months, any pre-existing neurological condition (including neurodegenerative disease, a primary diagnosis of ischemic or hemorrhagic stroke, or history of neurosurgery within the last 30 days)[12], less than 4 hours of sleep the night before [13], strenuous physical activity within 1 hours of testing [14], or any drug or alcohol use within the last 12 hours [15].

#### Participant recruitment

Different strategies were pursued depending on the location and the purpose. For the normative data, including the test-retest sample, participants from universities and high schools were referred by the athletic department, while those from community centers and the Texas Medical Center were recruited by posters in well-trafficked areas. Participants were unpaid, with the exception of a subset of the control adult participants in the Texas Medical Center who received a small compensation ($10) for taking sequential tests for the analysis of test-retest reliability.

For concussed patients and pain-matched controls, patients were recruited by nursing staff after diagnosis. BrainCheck’s concussion battery was always administered within 24 hours of the physician diagnosis. Pain matching relied on pain as self-reported to nurses via a research case report. Once identified, participants were then consented for participation in the experiment. Ten percent of non-concussed patients refused to participate, whereas thirty percent of concussed patients were eligible but refused to consent. The discrepancy is likely due to collateral symptoms like blurred vision or dizziness that affected the patients’ ability to use the tablet. These symptoms were more common in the concussed sample than the non-concussed, pain-matched sample. Both samples were infrequently interrupted during the administration of BrainCheck; however, they differed in terms of their willingness to restart the battery. Whereas the nonconcussed, pain-matched patient was always willing to restart, approximately 10% of concussed patients refused to restart. For the concussed group, 2 subjects had a GCS score of 14, while the others with the GCS score of 15. Seventy percent of the concussed patients had experienced loss of consciousness and amnesia. Patient demographics are shown in **Table 1**.

**Table 1:** Demographics of the study populations at the different types of test sites and for the different study populations.

	Mean age	Age ranges	% Male
**Site type**			
University	21.8	18–25	43%
High School	16.7	15–19	57%
Community Center (Children)	13.5	10–16	8%
Community Center (Adult)	36.1	18–64	44%
BrainCheck Office	47.5	25–64	48%
**Population**			
Healthy	24.9	10–64	46%
Concussed	32.2	23–63	53%
Control	33.6	22–59	40%

### Study design and setting

All participants completed the software battery using an iPad Air (model MD785LL/B) with a Wi-Fi connection. Data was collected over a 7-month period. Specifically, normative data was collected from November 19, 2015, until June 29, 2015. Test-retest data was collected from March 7, 2016, until June 17, 2016. Concussed and pain-matched control data was collected from March 1, 2016, until June 27, 2016.

All normative and test-retest data was collected by the same certified psychiatric professional, who holds a bachelor of science in exercise physiology from University of Houston. He has administered BrainCheck for two years in his capacity as clinical coordinator.

For the concussion and pain-matched controls, diagnoses came from several different ER physicians with an MD. Participants were identified by hospital nursing staff and then evaluated by one of the authors (SY), who was trained to use the BrainCheck battery to ensure consistency across settings.

### Methods and measurements

All participants (or their legal representatives) signed the informed consent forms prior to participation in the study, as approved by the Institutional Review Board at Baylor College of Medicine.

**Fig 1:**
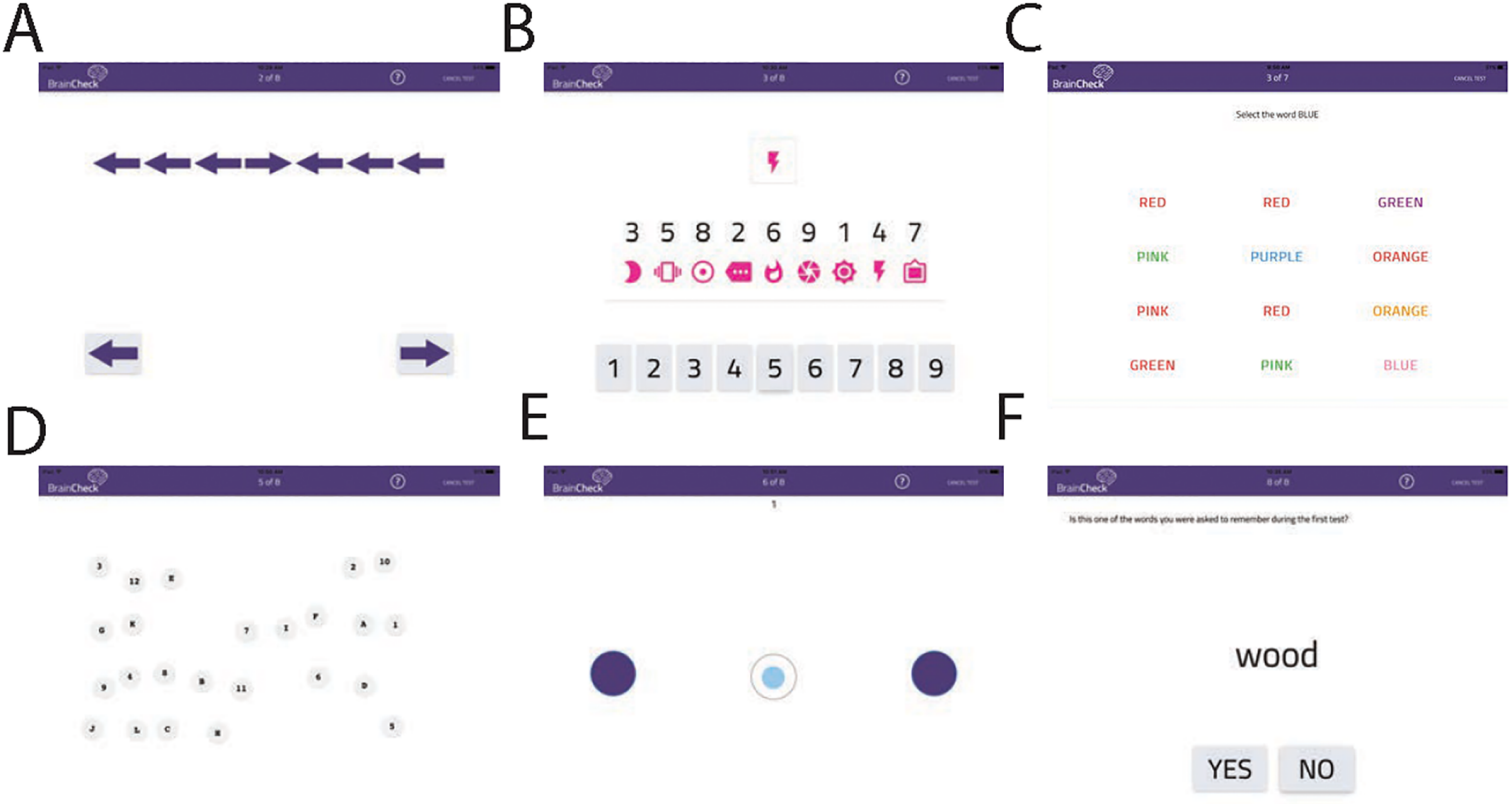
Screenshots of the 6 assessments in the BrainCheck battery.

We created tablet-based versions of six neurocognitive tests, described below (**Fig 1**); collectively, the battery of tests is referred to as "BrainCheck". We compared BrainCheck against the reference standard of a physician diagnosis of concussion. This choice mirrors the reference standard used in other studies of traumatic brain injury (see, e.g. [9]). A brief description of each neurocognitive test follows.

An effective test of attention is the *Flanker Task* [16]. Patients with mTBI show significantly longer reaction times for alerting, orienting and executive parts of attention when compared with normal controls [17]. The test presents participants with a target item (in this case, a central arrow) flanked by congruent or incongruent arrows. Participants identify the direction of the target as quickly and accurately as possible.

The *Digit Symbol Substitution Task* measures general cognitive performance. Participants must match an arbitrary correspondence of symbols to digits; when presented with a new symbol, they find as quickly as possible the corresponding digit and answer by pressing the digit. This is a continuous performance task in which the participant makes as many correct matches as possible within a fixed testing period. It has been previously demonstrated that mTBI patients perform significantly worse on this task than controls [18].

The *Stroop Task* measures the reaction time required to overcome cognitive interference [19]. When the name of a color (e.g., "blue," "green," or "red") is printed in an incongruent color (for example, the word "blue" printed in red), naming the color of the word takes longer and is more prone to errors than when the word and color are congruent. Commonly used as a measure of executive function, this task measures a subject’s ability to shift cognitive set [20] and provides a measure of cognitive inhibition [21, 22], that is, the ability to inhibit an overlearned response [20]. The Stroop task is often used to screen for brain damage [23]. The magnitude of "Stroop interference" (the difference in reaction times for congruent and non-congruent color words) is greater in patients with mTBI than the normal population [24]. The TBI Clinical trial network has demonstrated that the Stroop task has diagnostic value for identifying neurocognitive deficits in patients with TBI [25].

The *Trail Making Test (TMT)* is a neuropsychological test of visual attention and task switching. Participants are instructed to connect a set of 25 numbers in increasing order as rapidly as possible. The TMT provides measures of visual search speed, scanning, speed of processing, mental flexibility, and executive functioning. Trail Making Test A uses only numbers (1 through 25), while Trail Making Test B employs alternating letters and numbers (1 –A– 2 –B– 3 –C—…). Both TMT-A and TMT-B have strong positive predictive values as a diagnostic measure form TBI [26].

*Balance and coordination* are often impaired in patients with mTBI [27–30]. To detect subtle defects in that domain, we developed a test in which a ball is displayed on the tablet, moving according to the tilt of the tablet. A participant holds the tablet out in front at arm’s length, and tilts it appropriately to keep the ball in a central circle. The task is not difficult with normal coordination abilities; with a deficit it becomes measurably more difficult.

The *Immediate and Delayed Recall Tests* measure a participant’s ability to correctly recall seen words in the presence of distractors. First, immediate recall is measured by serially displaying 10 words, and then asking whether a word was just seen—either a distractor word or a target word (20 trials). At the end of the testing battery, without seeing the original list again, participants are again presented with 20 words and asked whether each word was presented before. Both immediate and delayed recall show sensitivity to mTBI [31].

### Analysis

We analyzed the data using custom software written in MATLAB. A group of tests taken by a single user at a single time is defined as a battery. We considered only complete batteries and, for the control populations, removed outlier data that was more than 3 standard deviations from the mean. Principle component analysis (PCA) was performed with the MATLAB function ‘pca’, which is built into the statistics and machine learning toolbox. Statistical significance of differences in mean values between groups was evaluated using the two-sample t-test, while statistical significance for different distributions was determined using the two-sample Kologorov-Smirnov test. All figure error bars reflect standard deviation from bootstrapping. Data is available at https://figshare.com/s/352b64af1ca84ed9251d (doi: 10.6084/m9.figshare.4887314).

## Results

### Characteristics of study subjects

We enrolled 30 patients in the mTBI group (53% males, median age 32.2), 30 users in the pain-matched control group (40% males, median age 33.6), and 538 participants in the healthy control group (46% males, median age 24.9). Demographics are presented in **Table 1**. The battery of all six tests was completed within five minutes by the majority of users.

### Main results: Normative data

**Fig 2:**
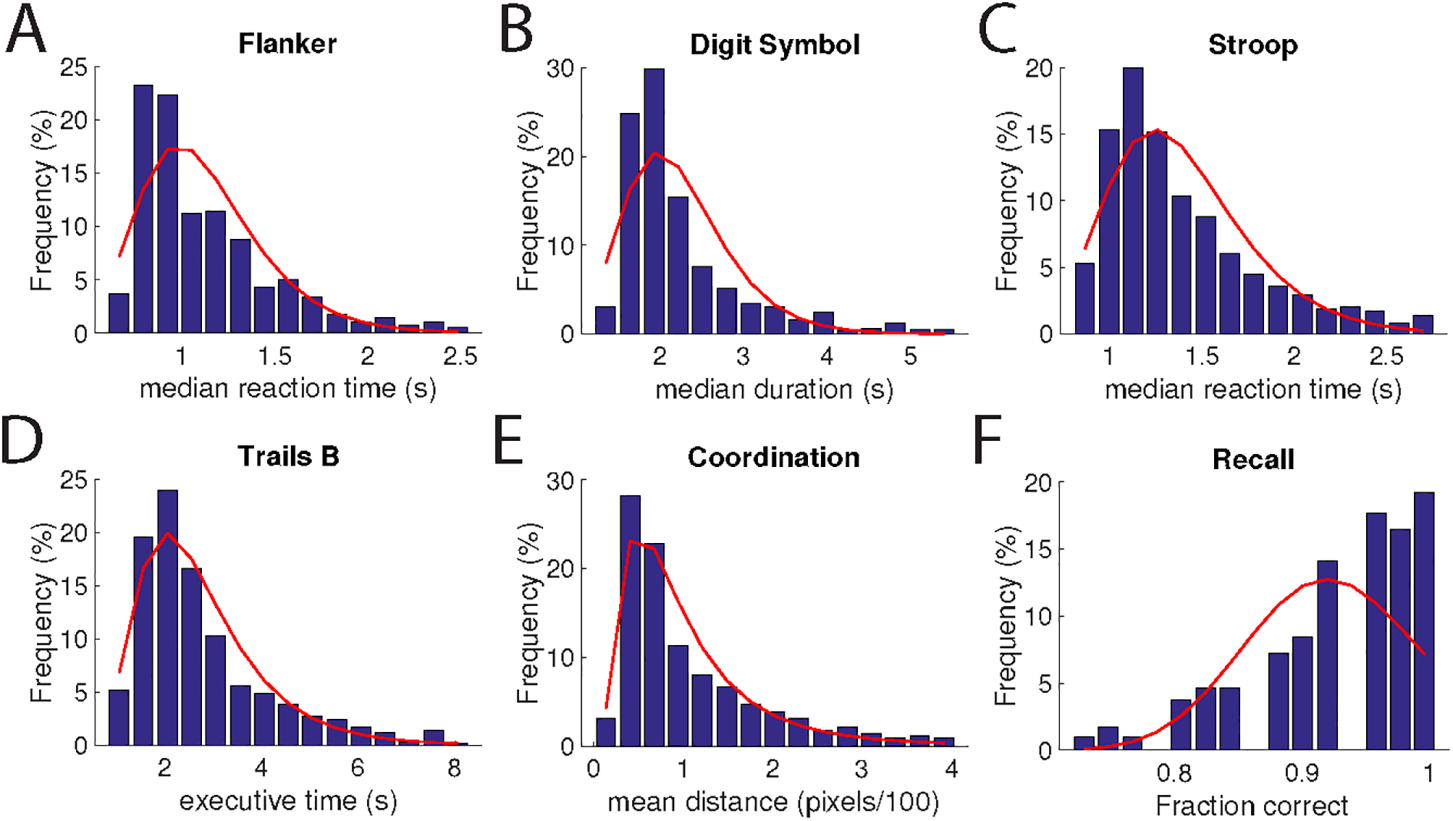
Normative data. **Each panel shows data from the indicated assessment.** Only data from healthy individuals was used to create these histograms.

Normative data for all the tests using only the data from the healthy control group is shown in **Fig 2** and **Table 2**. In general, the data were well approximated by log-normal distributions, and with sufficient samples size such that the standard error of the means were quite small (1–2% of the mean values).

**Table 2:** Statistics of assessments included in the BrainCheck battery.

Measure	Mean	Standard Dev	Standard Error
1. Flanker	1.14	0.38	0.02
2. Digit Symbol Substitution	2.28	0.76	0.03
3. Stroop	1.62	0.54	0.02
4. Trail Making (A)	1.51	0.63	0.03
5. Trail Making (B)	2.81	1.38	0.05
6. Coordination	1.12	0.83	0.04
7. Recall	0.92	0.07	0.003
8. Ebbinghaus Illusion	-0.24	2.37	0.10

#### Differences in test performance by age and sex

**Fig 3:**
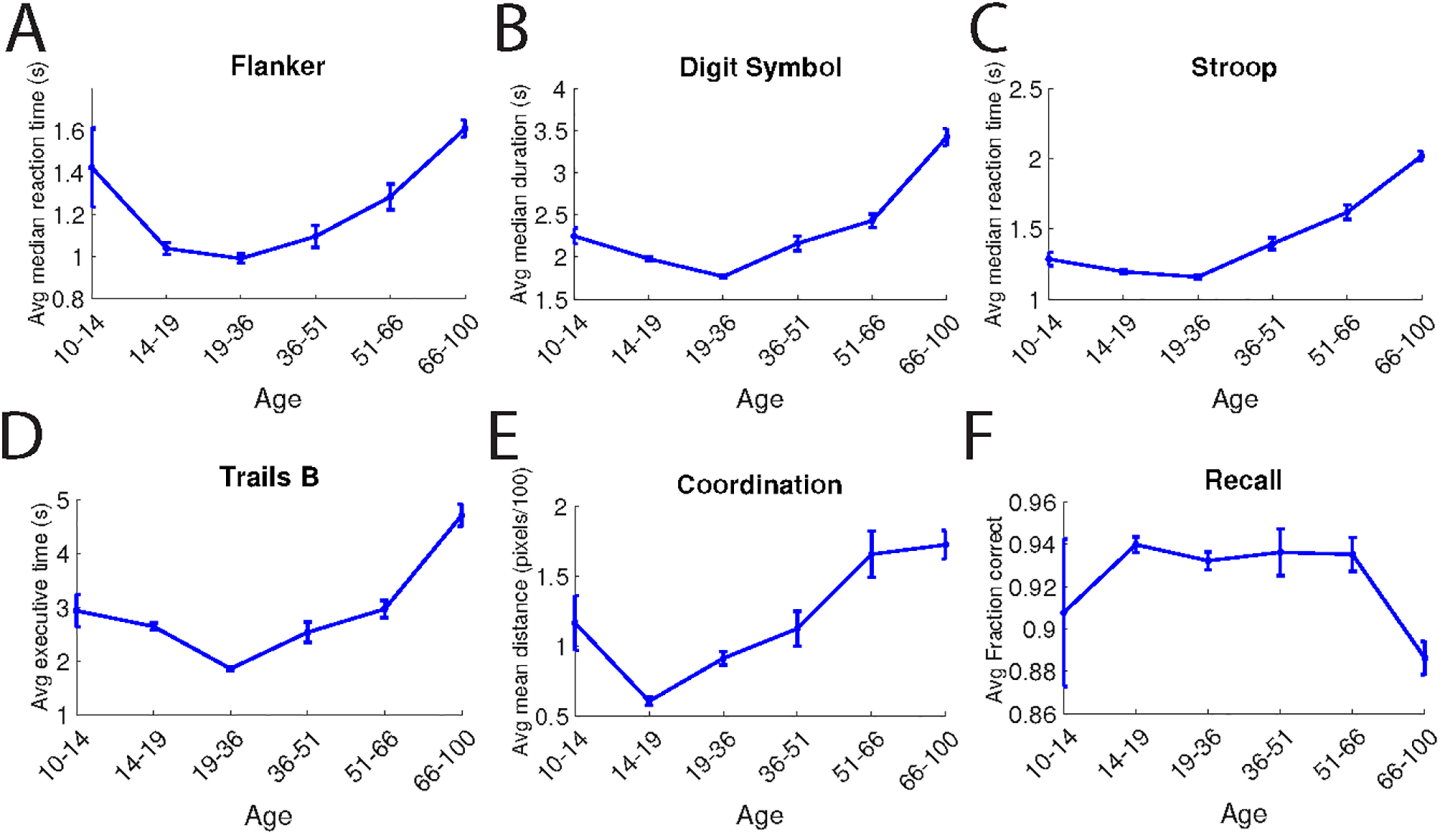
Age dependence of the BrainCheck assessments. For each battery, data shows the mean value of the indicated age ranges. Error bars indicate standard error of the mean, computed by bootstrapping.

**Fig 4:**
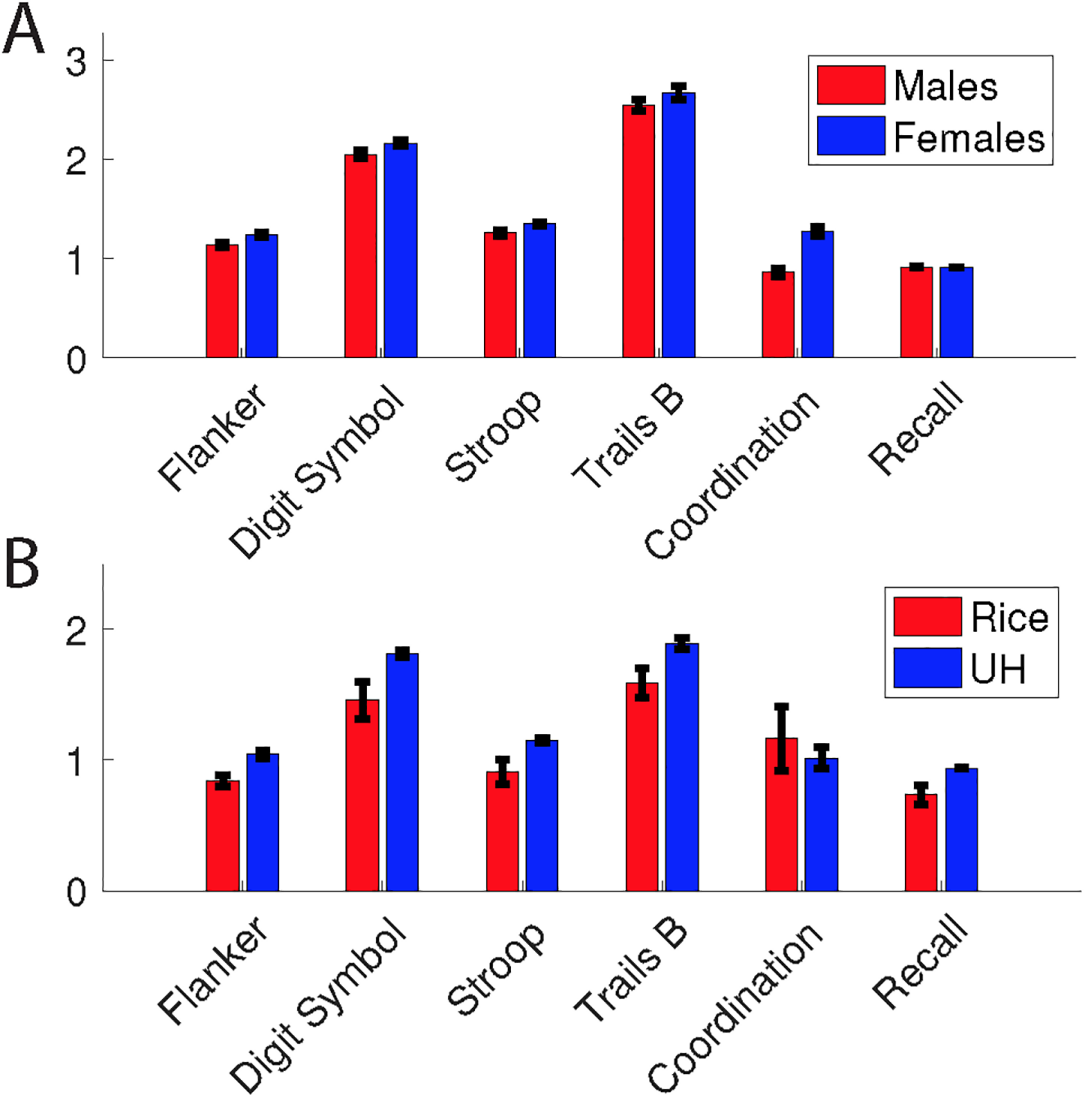
**Performance on BrainCheck assessments by (A) gender and (B) socioeconomic status.** As explained in the text, assessments performed at different university test sites were used as a rough measure of the effect of socioeconomic status. Error bars represent standard error of the mean, computed by bootstrapping.

All tests showed age-dependent performance differences, with peak performance in the population aged between 19 and 51 and declining in older or younger cohorts (**Fig 3**). Although there were differences between the age brackets, there were no performance differences by sex (**Fig 4**), with the exception of small performance advantages for males on the coordination test (p < 0.02) and for females on the Trail Making Test-B (p < 1 × 10−5).

#### Test-retest reliability

**Fig 5:**
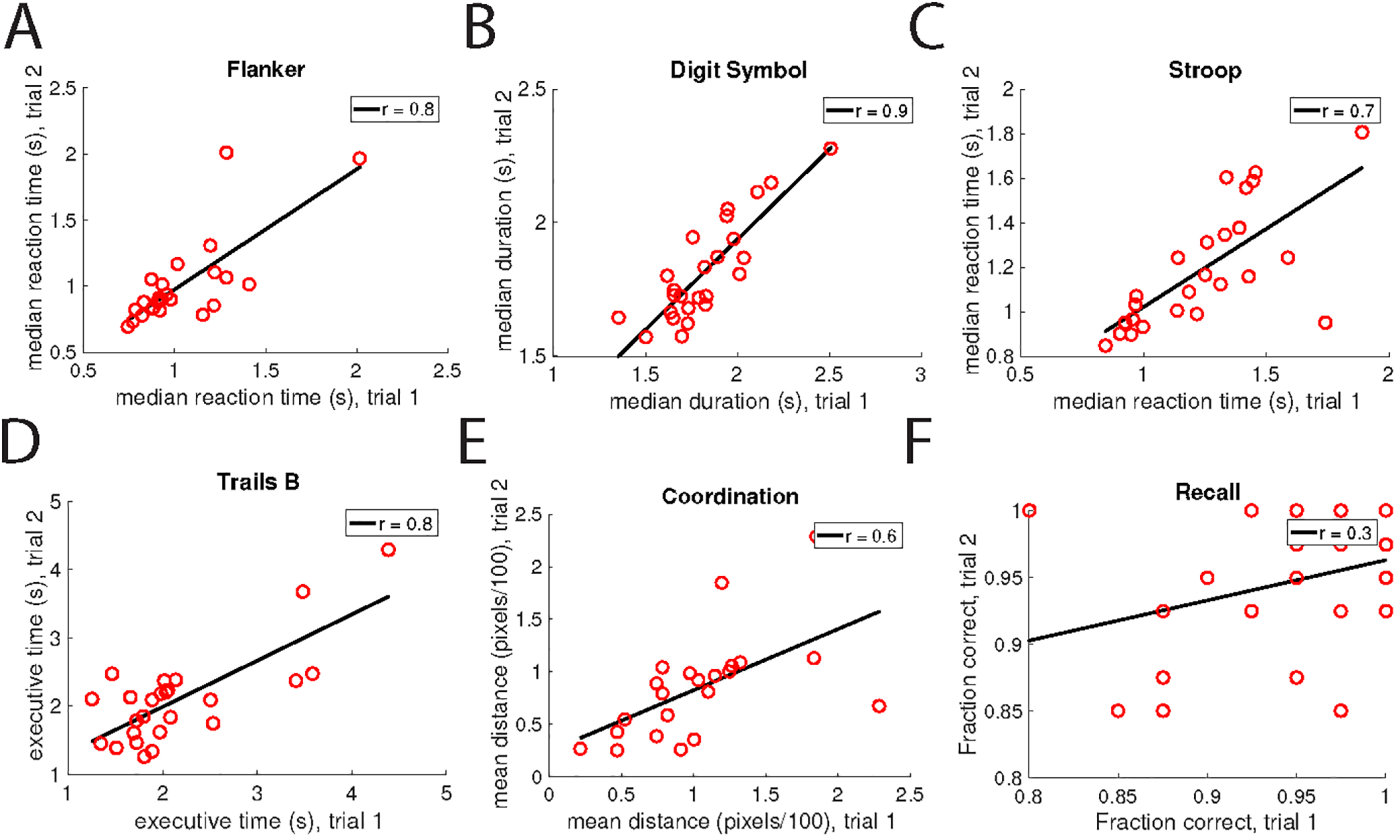
Test-retest reliability. In all panels, each datapoint represents an individual who took the same assessment on two different dates. Black lines represent linear fits to the data. r-values for the fits are shown in the legend of each panel.

We next sought to determine the reliability of the tests by comparing the results from users who took the assessment more than once. The time interval between test administrations was at least 7 days. We found that most individual tests showed strong retest reliability with correlation coefficients between first and second trials ranging from 0.6 to 0.9, as shown in **Fig 5**.

### Main results: Performance of concussed individuals

**Fig 6:**
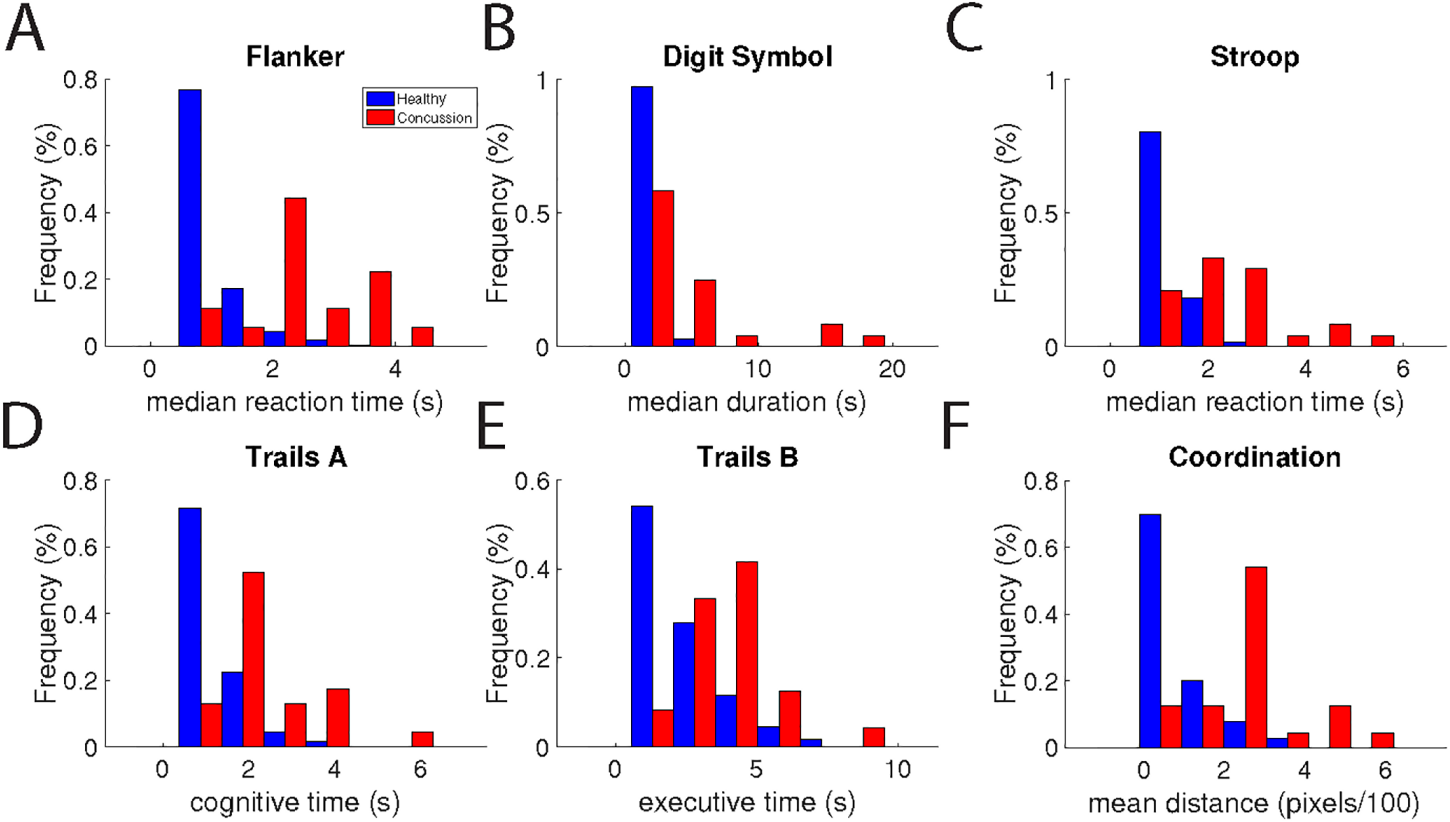
Individual metrics differ for concussed and healthy individuals. For each assessment, normative histograms for healthy (blue) or concussed (red) individuals are shown.

The performance of 30 mTBI patients was compared to 30 orthopedic controls. All tests were capable of distinguishing mTBI from controls (**Fig 6**) and mTBI from healthy individuals (see **Table 3** for p-values of the differences in the means of these groups for each test).

**Table 3:** Performance of 30 mTBI patients compared to 30 orthopedic controls.

Measure	concussion vs. control	concussion vs. healthy
1. Flanker	7.9 × 10^−4^	1.2 × 10^−52^
2. Digit Symbol Substitution	4.3 × 10^−4^	4.6 × 10^−38^
3. Stroop	5.1 × 10^−6^	4.1 × 10^−39^
4. Trail Making (A)	3.3 × 10^−4^	8.5 × 10^−23^
5. Trail Making (B)	2.5 × 10^−3^	5.7 × 10^−4^
6. Coordination	5.5 × 10^−3^	1.5 × 10^−23^
7. Recall	1.2 x 10^−4^	2.4 x 10^−24^

**Fig 7:**
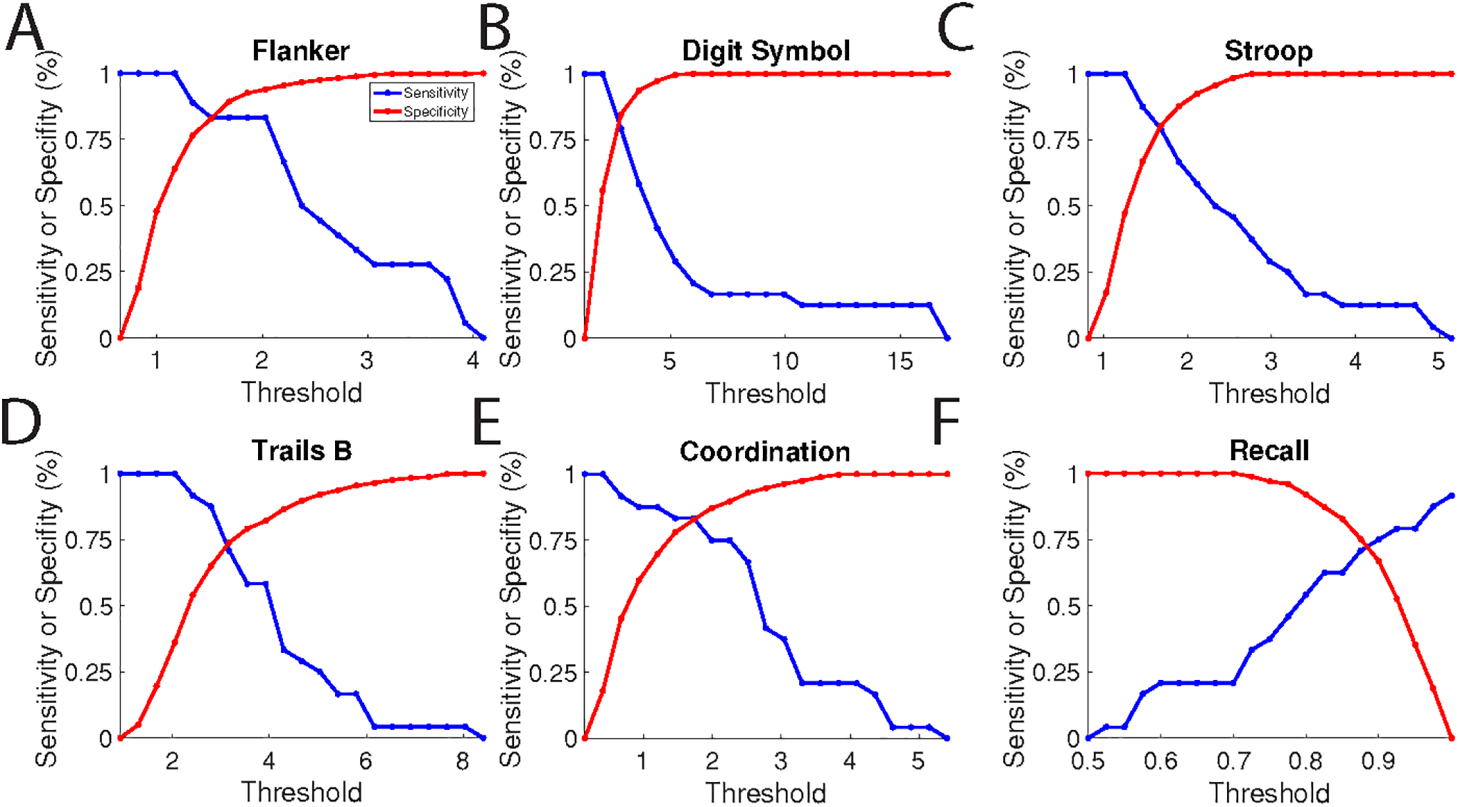
Sensitivity and specificity of individual assessments. For each assessment the sensitivity (true positive rate; red) and specificity (true negative rate; blue) are plotted as a function of the threshold for discriminating concussed from healthy individuals.

We also examined the performance of the individual tests as diagnostic of mTBI, presented as specificity and sensitivity as a function of the threshold for distinguishing TBI and healthy individuals (**Fig 7**). The most specific tests resulted in slightly above 50% detection, while the most sensitive test (the coordination test, 77% sensitivity), yielded 75% specificity.

#### Defining a scoring metric for concussions

**Fig 8:**
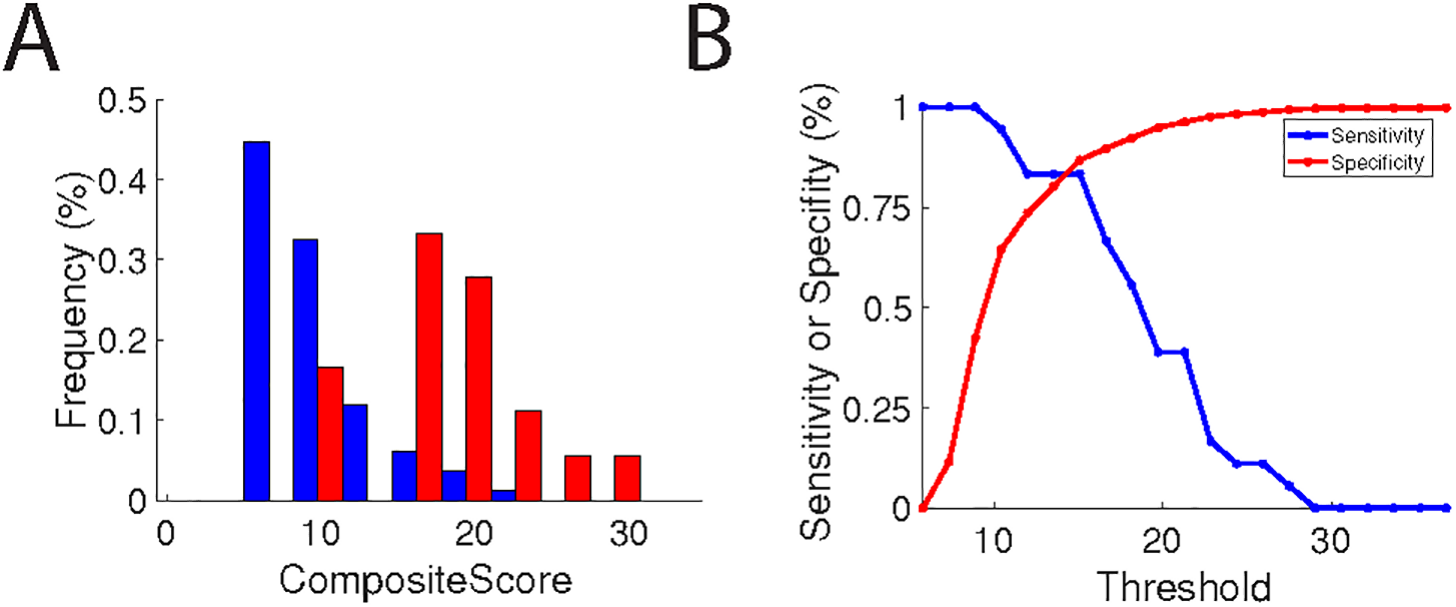
A composite score distinguishes concussed and healthy individuals. (A) Distribution of the composite score for healthy (blue) and concussed (red) individuals. (B) Sensitivity (red) and specificity (blue) of a test based on the composite score plotted as a function of the threshold for identifying concussed individuals.

To maximize the sensitivity and specificity of the individual tests, we sought to define a combined metric which would robustly discriminate patients with TBI while minimizing false positives. Thus, we defined an optimized linear sum of the scores from all six assessments. The mean of this score differed significantly between concussed and control individuals (p < 3×10−5), and between concussed and healthy individuals (p < 1×10−20) (**Fig 8**). This metric also provided a sensitive and specific test for TBI with sensitivity and specificity of 83% and 87%, respectively (**Fig 8**).

#### An independent test for malingering

**Fig 9:**
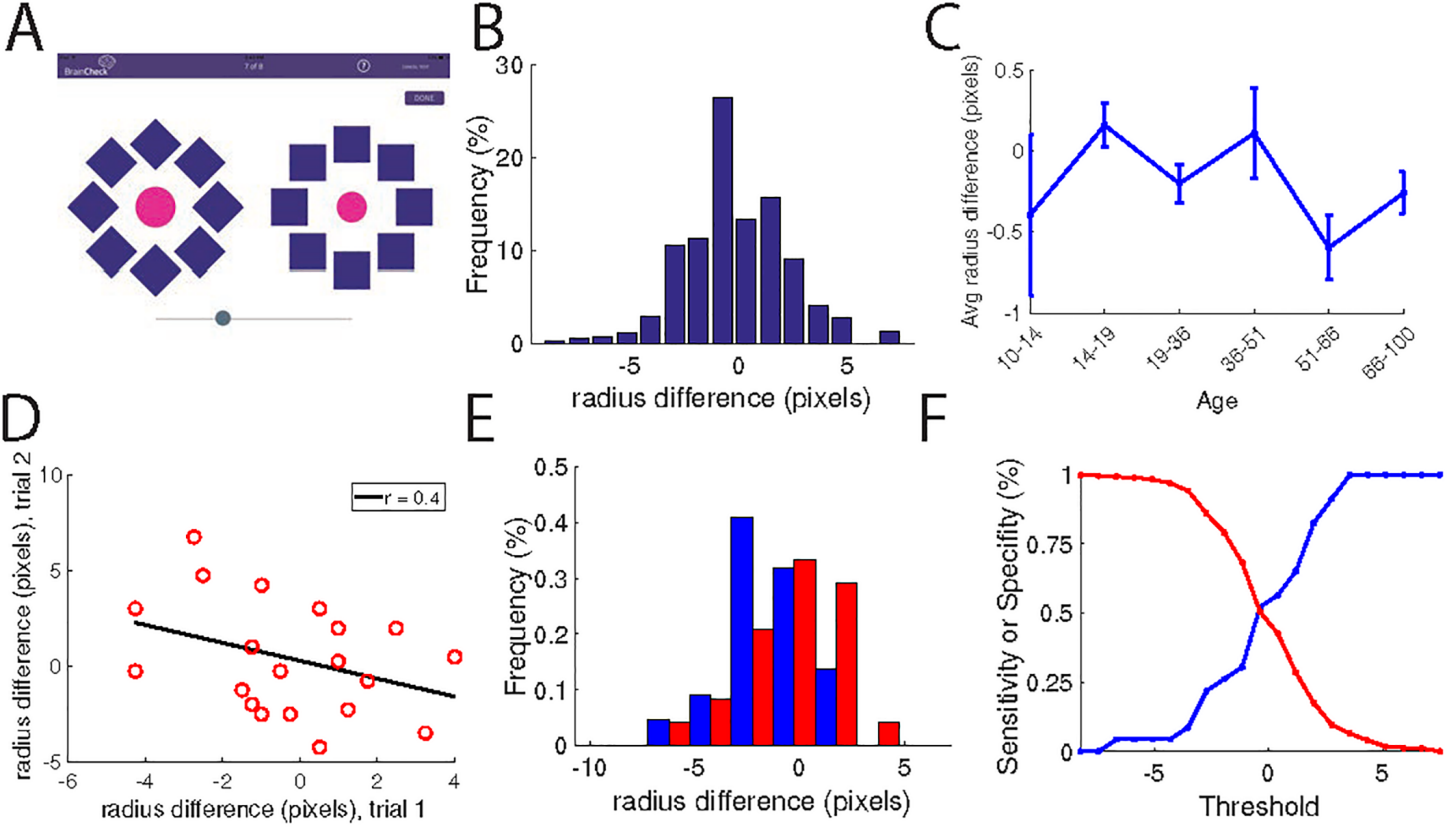
A test for malingering shows no dependence on cognitive performance. (A) Screenshot of the malingering test. (B) Normative data for the test (C) Scores on the malingering test plotted by age. (D) Data for individuals who took the malingering test twice separated by at least one week. The score on the first trial is plotted against the score on the second. (E) Distribution of scores on the malingering test for healthy (blue) and concussed (red) individuals. (F) Specificity (blue) and sensitivity (red) of a test to distinguish concussed and healthy individuals based on the malingering test.

Many test takers may have incentives to intentionally perform poorly. Athletes who perform poorly at baseline could potentially more easily return to play following an incident, while others may deliberately perform poorly on a post-incident assessment in order to influence their diagnosis for insurance purposes. As a hidden metric for malingering, we included a test to detect deliberate poor performance in the battery. We asked participants to use a slider to adjust the relative size of two circles until they appeared to be equal (**Fig 9**). If the circles are surrounded by shapes of the same size, participants should be able to make the circles equally sized, even if the person has experienced an mTBI; the only way a person could ‘fail’ the test is if he were deliberately trying to do so. This test showed normally distributed data around the expected mean of zero pixels. Most importantly, concussed and healthy individuals performed similarly on the test so a large outlier performance on this test presumably indicates malingering rather than mTBI.

## Discussion

Patients with mTBI typically present with subtle deficits in perception, attention, executive function, working memory, spatial attention, and coordination—all of which can be detected with simple tests. While any one of these tests can suggest mTBI, the combination of six tests creates a much more comprehensive tool and thus has more opportunity to detect mTBI. In this study, we demonstrated that a simple battery of tablet-based, easy to understand psychophysical tests has high sensitivity and specificity for detecting mTBI.

Other computer-based psychophysics tests for mTBI exist, such as the ImPACT test, which has approximately the same specificity and sensitivity (89% and 82%, respectively) as BrainCheck [32, 33]. However, the advantage of the current tests is that they are briefer, tablet-based, gamified, and very straightforward to use. The ability to perform the BrainCheck battery on a tablet in approximately five minutes (as compared to over 30 minutes for the ImPACT test) suggests an easier adoption into athletics, emergency situations, and the military.

Many other detection techniques are currently in development. This includes the assessment of serum biomarkers, quantitative EEG, smooth pursuit eye tracking, and a variety of imaging techniques (e.g., diffusion tensor imaging, high definition fiber tractography, and magnetic resonance spectroscopy) but these approaches are expensive, require expertise to administer, and have an unclear future for use on the field. This leaves a need for first responders to have a decision-assist device in the field—one that is portable, accurate, and requires no expertise to administer. Tablet computing makes a portable approach to mTBI measurement possible and it may offer characteristics not available with other types of objective testing. Further, because this test can be performed rapidly, with no expertise to administer, and has safeguards to preclude malingering, it may be a candidate for use by military and athletics teams to rapidly make fitness-for-duty or return-to-play decisions.

As with all tests, there is a trade-off between sensitivity and specificity. This tradeoff is shown explicitly for the individual tests in **Fig 7** and for the composite score in **Fig 8**. Considering the nature of TBI, it may be useful to have a test that is highly sensitive, even if it is not highly specific, or highly specific, even if it is not highly sensitive. For example, when making return-to-play decisions, a "better safe than sorry" approach is in order, making sensitivity the most important parameter. By choosing a low threshold (around 10), BrainCheck tests can achieve nearly 100% sensitivity with a reduction in specificity to approximately 70%. Conversely, in emergency department settings, CT scans are typically ordered, even for minor of head injuries. By choosing a high threshold (around 22), BrainCheck can achieve nearly 100% specificity, while reducing sensitivity to approximately 30%. Because even a small reduction in unnecessary CT scans would be beneficial, it may be useful to have a test that can be optimized for specificity. By tuning the threshold, BrainCheck testing application provides a rapid, portable testing method for concussion that could be a very effective first line test for a wide variety of settings.

## References

[1] Daneshvar DH, Nowinski CJ, McKee AC, Cantu RC. The epidemiology of sport-related concussion. Clin Sports Med. 2011; 30(1):1–17. doi: 10.1016/j.csm.2010.08.006 21074078 10.1016/j.csm.2010.08.006 PMC2987636

[2] Control CfD, Prevention U. Nonfatal traumatic brain injuries related to sports and recreation activities among persons aged ≤19 years—United States, 2001–2009. MMWR Morb Mortal Wkly Rep. 2011; 60(39):1337–42. 21976115

[3] Lew HL, Thomander D, Chew KT, Bleiberg J. Review of sports-related concussion: Potential for application in military settings. J Rehabil Res Dev. 2007; 44(7):963–74. Epub 2007/12/14. 18075953 10.1682/jrrd.2006.12.0169

[4] Greenwald RM, Chu JJ, Beckwith JG, Crisco JJ. A proposed method to reduce underreporting of brain injury in sports. Clin J Sport Med. 2012; 22(2):83–5. doi: 10.1097/JSM.0b013e31824cc5d3 22388342 10.1097/JSM.0b013e31824cc5d3

[5] Halstead ME, Walter KD. Sport-related concussion in children and adolescents. Pediatrics. 2010; 126(3):597–615. doi: 10.1542/peds.2010-2005 20805152 10.1542/peds.2010-2005

[6] Lovell MR, Fazio V. Concussion management in the child and adolescent athlete. Curr Sports Med Rep. 2008; 7(1):12–5. doi: 10.1097/01.CSMR.0000308671.45558.e2 18296938 10.1097/01.CSMR.0000308671.45558.e2

[7] Rivara FP, Graham R. Sports-related concussions in youth: report from the Institute of Medicine and National Research Council. JAMA. 2014; 311(3):239–40. Epub 2013/11/05. doi: 10.1001/jama.2013.282985 24185195 10.1001/jama.2013.282985

[8] Arciniegas DB, Anderson CA, Topkoff J, McAllister TW. Mild traumatic brain injury: a neuropsychiatric approach to diagnosis, evaluation, and treatment. Neuropsychiatr Dis Treat. 2005; 1(4):311–27. 18568112 PMC2424119

[9] Giza CC, Kutcher JS, Ashwal S, Barth J, Getchius TSD, Gioia GA, et al Summary of evidence-based guideline update: evaluation and management of concussion in sports: report of the Guideline Development Subcommittee of the American Academy of Neurology. Neurology. 2013; 80(24):2250–7. doi: 10.1212/WNL.0b013e31828d57dd 23508730 10.1212/WNL.0b013e31828d57dd PMC3721093

[10] Kontos AP, Sufrinko A, Womble M, Kegel N. Neuropsychological assessment following concussion: an evidence‐based review of the role of neuropsychological assessment pre- and post-concussion. Curr Pain Headache Rep. 2016; 20(6):38 doi: 10.1007/s11916-016-0571-y 27099226 10.1007/s11916-016-0571-y

[11] Jagoda AS, Bazarian JJ, Bruns JJ Jr, Cantrill SV, Gean AD, Howard PK, et al Clinical policy: neuroimaging and decisionmaking in adult mild traumatic brain injury in the acute setting. Ann Emergency Med. 2008; 52(6):714–48. doi: 10.1016/j.annemergmed.2008.08.021 19027497 10.1016/j.annemergmed.2008.08.021

[12] Ricard C, Casez P, Gstalder H, Mawazini S, Gauthier V, Fontanel A, et al Six-month outcome of 795 patients admitted to Annecy hospital emergency department for mild traumatic brain injury. Sante Publique (Bucur). 2013; 25(6):711–8. Epub 2014/01/24. 24451416

[13] Berg DB, Engel AM, Saba A, Hatton EK. Differences in Public Belief and Reality in the Care of Operative Patients in a Teaching Hospital. J Surg Educ. 2011; 68(1):10–8. doi: 10.1016/j.jsurg.2010.08.005 21292209 10.1016/j.jsurg.2010.08.005

[14] Ortega E. Neuroendocrine mediators in the modulation of phagocytosis by exercise: physiological implications. Exerc Immunol Rev. 2003; 9:70–93. Epub 2003/12/23. 14686096

[15] Miller P, Droste N, Baker T, Gervis C. Last drinks: A study of rural emergency department data collection to identify and target community alcohol-related violence. Emerg Med Australas. 2015; 27(3):225–31. doi: 10.1111/1742-6723.12369 25720820 10.1111/1742-6723.12369

[16] Fan J, Flombaum JI, McCandliss BD, Thomas KM, Posner MI. Cognitive and brain consequences of conflict. NeuroImage. 2003; 18(1):42–57. doi: 10.1006/nimg.2002.1319 12507442 10.1006/nimg.2002.1319

[17] van Donkelaar P, Langan J, Rodriguez E, Drew A, Halterman C, Osternig LR, et al Attentional deficits in concussion. Brain Inj. 2005; 19(12):1031–9. doi: 10.1080/02699050500110363 16263646 10.1080/02699050500110363

[18] De Monte VE, Geffen GM, May CR, McFarland K. Improved sensitivity of the rapid screen of mild traumatic brain injury. J Clin Exp Neuropsychol. 2010; 32(1):28–37. doi: 10.1080/13803390902806519 19418329 10.1080/13803390902806519

[19] Melara RD, Algom D. Driven by information: a tectonic theory of Stroop effects. Psychol Rev. 2003; 110(3):422–71. doi: 10.1037/0033-295x.110.3.422 12885110 10.1037/0033-295x.110.3.422

[20] Strauss E, Sherman EMS, Spreen O. A compendium of neuropsychological tests: Administration, norms, and commentary 3rd ed. New York: Oxford University Press; 2006.

[21] Archibald SJ, Kerns KA. Identification and description of new tests of executive functioning in children. Child Neuropsychol. 1999; 5(2):115–29. doi: 10.1076/chin.5.2.115.3167

[22] Boone KB, Miller BL, Lesser IM, Hill E, D'Elia L. Performance on frontal lobe tests in healthy, older individuals. Dev Neuropsychol. 1990; 6(3):215–23. doi: 10.1080/87565649009540462

[23] Homack S, Riccio CA. A meta-analysis of the sensitivity and specificity of the Stroop Color and Word Test with children. Arch Clin Neuropsychol. 2004; 19(6):725–43. doi: 10.1016/j.acn.2003.09.003 15288327 10.1016/j.acn.2003.09.003

[24] MacLeod CM. Half a century of research on the Stroop effect: An integrative review. Psychol Bull. 1991; 109(2):163–203. doi: 10.1037/0033-2909.109.2.163 2034749 10.1037/0033-2909.109.2.163

[25] Bagiella E, Novack TA, Ansel B, Diaz-Arrastia R, Dikmen S, Hart T, et al Measuring outcome in traumatic brain injury treatment trials: recommendations from the traumatic brain injury clinical trials network. J Head Trauma Rehabil. 2010; 25(5):375–82. doi: 10.1097/HTR.0b013e3181d27fe3 20216459 10.1097/HTR.0b013e3181d27fe3 PMC2939167

[26] Cicerone KD, Azulay J. Diagnostic utility of attention measures in postconcussion syndrome. Clin Neuropsychol. 2002; 16(3):280–9. doi: 10.1076/clin.16.3.280.13849 12607141 10.1076/clin.16.3.280.13849

[27] Greenwald BD, Cifu DX, Marwitz JH, Enders LJ, Brown AW, Englander JS, et al Factors associated with balance deficits on admission to rehabilitation after traumatic brain injury: a multicenter analysis. J Head Trauma Rehabil. 2001; 16(3):238–52. doi: 10.1097/00001199-200106000-00003 11346446 10.1097/00001199-200106000-00003

[28] Rinne MB, Pasanen ME, Vartiainen MV, Lehto TM, Sarajuuri JM, Alaranta HT. Motor performance in physically well-recovered men with traumatic brain injury. J Rehabil Med. 2006; 38(4):224–9. doi: 10.1080/16501970600582989 16801204 10.1080/16501970600582989

[29] Kaufman KR, Brey RH, Chou L-S, Rabatin A, Brown AW, Basford JR. Comparison of subjective and objective measurements of balance disorders following traumatic brain injury. Med Eng Phys. 2006; 28(3):234–9. doi: 10.1016/j.medengphy.2005.05.005 16043377 10.1016/j.medengphy.2005.05.005

[30] Geurts ACH, Ribbers GM, Knoop JA, van Limbeek J. Identification of static and dynamic postural instability following traumatic brain injury. Arch Phys Med Rehabil. 1996; 77(7):639–44. doi: 10.1016/S0003-9993(96)90001-5 8669988 10.1016/s0003-9993(96)90001-5

[31] Comerford VE, Geffen GM, May C, Medland SE, Geffen LB. A Rapid Screen of the Severity of Mild Traumatic Brain Injury. J Clin Exp Neuropsychol. 2002; 24(4):409–19. doi: 10.1076/jcen.24.4.409.1044 12187455 10.1076/jcen.24.4.409.1044

[32] Schatz P, Pardini JE, Lovell MR, Collins MW, Podell K. Sensitivity and specificity of the ImPACT Test Battery for concussion in athletes. Arch Clin Neuropsychol. 2006; 21(1):91–9. doi: 10.1016/j.acn.2005.08.001 16143492 10.1016/j.acn.2005.08.001

[33] Nelson LD, Pfaller AY, Rein LE, McCrea MA. Rates and Predictors of Invalid Baseline Test Performance in High School and Collegiate Athletes for 3 Computerized Neurocognitive Tests. The American Journal of Sports Medicine. 2015; 43(8):2018–26. doi: 10.1177/0363546515587714 26059178 10.1177/0363546515587714 PMC4747101

